# Clinical Features and Survival Outcome in Aggressive-Type Adult T-Cell Leukemia/Lymphoma Patients: Real-Life Experience of a Single Center from an HTLV-1 Endemic Country

**DOI:** 10.3390/medicina60060872

**Published:** 2024-05-26

**Authors:** Iuliana Iordan, Ana-Maria Vlădăreanu, Cristina Mambet, Minodora Onisâi, Diana Cîșleanu, Horia Bumbea

**Affiliations:** 1 Department of Hematology, “Carol Davila” University of Medicine and Pharmacy, Emergency University Hospital of Bucharest, 050098 Bucharest, Romania; iuliana.iordan@drd.umfcd.ro (I.I.);; 2 Department of Medical Semiology and Nephrology, “Carol Davila” University of Medicine and Pharmacy, 050474 Bucharest, Romania; 3 Department of Virology, “Stefan S. Nicolau” Institute, 030304 Bucharest, Romania

**Keywords:** adult T-cell leukemia/lymphoma, ATLL, aggressive-type ATLL, human T-cell lymphotropic virus type I, HTLV-1

## Abstract

*Background and Objectives*: Adult T-cell leukemia/lymphoma (ATLL) is a highly aggressive T-cell lymphoproliferative disease associated with the human T-cell lymphotropic virus type I (HTLV-1). ATLL is a rare disease, found more frequently in HTLV-1-endemic areas, Romania being one of them. Despite treatment advances, the prognosis remains dismal. We aimed to describe the clinical, biological, and survival outcome features of Romanian patients with aggressive-type ATLL. *Materials and Methods:* We report the data of a prospective, observational, and unicentric study of all 20 patients diagnosed with lymphoma and acute types of ATLL at our center over the past 12 years. Data were collected from the patients’ medical records. *Results*: Lymphoma-type ATLL (60%) was more common than acute-type ATLL (40%). Median age at diagnosis was 40.5 years, and most patients were female. Laboratory data revealed significant differences between acute and lymphoma-type ATLL, namely, higher leukocyte (*p* = 0.02) and lymphocyte counts (*p* = 0.02) and higher levels of corrected calcium (*p* = 0.001) in acute-type ATLL. All patients received chemotherapy, and only two underwent allogeneic stem cell transplantation. Only six patients obtained a complete or partial response to chemotherapy, mostly the lymphoma-type ones. The median survival for all patients was 6.37 months, with higher survival in the lymphoma-type ATLL (8.16 months) than in the acute-type (3.60 months). Normal calcium levels (*p* = 0.011), uric acid (*p* = 0.005), BUN score (*p* = 0.000), JCOG-PI moderate risk (*p* = 0.038), and obtaining complete or partial response (*p* = 0.037) were associated with higher survival. *Conclusion*: Aggressive-type ATLL among Romanian patients presents distinct characteristics, including younger age at diagnosis, female predominance, and higher incidence of lymphoma-type ATLL compared to currently reported data. Survival remains very low, with all subtypes experiencing a median survival of less than one year.

## 1. Introduction

Adult T-cell leukemia/lymphoma (ATLL) is a rare and very aggressive T-cell neoplasm characterized by the proliferation of peripheral T-cells, described for the first time in 1977 in Japan [1]. ATLL is associated with human T-cell lymphotropic virus type I (HTLV-1) infection, which is endemic in Japan, the Caribbean Islands, Central and South America, tropical Africa, central Australia, and Romania [2,3].

Adult T-cell leukemia/lymphoma occurs in only 3–5% of carriers, after a latency of 20 to 30 years [4]. The mechanisms that lead to disease development in carriers are currently poorly understood. A high proviral load (PVL) is associated with a higher risk of developing ATLL [5,6]. Among the carriers who had a PVL greater than four copies/100 peripheral blood mononuclear cells (PBMc), 25% progressed to ATLL, while none of those who had a lower PVL progressed to ATLL [5]. The high PVL is sustained by persistent clonal proliferation and increased survival of the HTLV-1-infected cells. Over time, aggressive clones are selected due to gene mutations and epigenetic changes that grant them a growth advantage. It was demonstrated that these clones emerged years before the onset of ATLL [7]. Viral proteins HTLV-1 basic leucine zipper factor (HBZ) and Tax are essential for leukemogenesis, promoting viral persistence, proliferation of the HTLV-1-infected cells, and immune and inflammatory responses [6,8]. The effects of viral proteins also influence cytokine production. In ATLL, the cytokine profile promotes tumor proliferation and facilitates the escape from the immune response [9].

Adult T-cell leukemia/lymphoma is classified by Shimoyama et al. into four subtypes based on clinical and biological characteristics, namely, the presence of lymphadenopathies, the type of extranodal infiltration, the absolute lymphocyte count, the percentage of atypical lymphocytes in the peripheral blood, serum calcium, and lactate dehydrogenase (LDH) [10]. The smoldering and favorable chronic ones are considered indolent subtypes, whereas the chronic unfavorable, lymphoma, and acute ones are aggressive subtypes [10].

The clinical evolution of ATLL is marked by multiple complications as a result of disease progression or treatment. Most common ones are bone marrow suppression, compression syndrome by large lymphadenopathies, extranodal infiltrations, tumor lysis syndrome, hypercalcemia, severe and opportunistic infections because of immunosuppression, and neurological complications [11,12,13,14,15].

Treatment options outside clinical trials include zidovudine/interferon, conventional chemotherapy, monoclonal antibodies (the defucosylated humanized anti-CCR4 monoclonal antibody—Mogamulizumab), and early up-front allogeneic stem cell transplantation for eligible patients [16]. However, despite more recent advances in treatment and supportive care, the prognosis of ATLL patients remains dismal. The treatment choices remain limited, and only a small proportion of patients with aggressive subtypes obtain long-term survival [17].

## 2. Materials and Methods

We conducted a prospective, observational, and unicentric study of all 20 patients diagnosed with ATLL at the Emergency University Hospital of Bucharest between January 2011 and December 2023. All patients had aggressive forms of ATLL. The diagnosis relied on HTLV-1 seropositivity in conjunction with histology, cytology, or immunophenotype. Patients’ data were obtained from the available medical records. We documented epidemiological data, disease type and extent at diagnosis, clinical status at diagnosis, baseline biological parameters, treatment modalities, and outcomes.

The patients were classified according to Shimoyama criteria [10] and the Lugano staging system [18]. All patients with acute-type ATLL were characterized as stage IV by definition because of the peripheral blood involvement. We were unable to calculate the acute- and lymphoma-type ATLL Prognostic Index (ATL-PI) or other prognosis scores based on molecular genetics due to the lack of accessibility to the soluble IL-2 receptor analysis and molecular exam. Therefore, we used the Japan Clinical Oncology Group prognostic index (JCOG-PI), which includes corrected calcium levels and performance status. JCOG-PI identifies two risk groups: moderate-risk (corrected calcium < 2.75 mmol/L and performance status < 2) and high-risk (one or both risk factors) [19].

All patients received multiagent chemotherapy. We used the following regimens as first or subsequent lines of treatment: CHOP (cyclophosphamide, doxorubicin, vincristine, prednisone), CHOP-like regimens, modified LSG15 (vincristine instead of vindesine, without ranimustine—unavailable in our country), hyper-CVAD (cycle A: hyper-fractionated cyclophosphamide, vincristine, doxorubicin, and prednisolone; cycle B: high-dose methotrexate and cytarabine), GEMOx (gemcitabine, oxaliplatin), DHAOx (dexamethasone, cytarabine, oxaliplatin), DHAP (dexamethasone, cytarabine, cisplatin).

The number of lines of therapy ranged from one to four. All patients received first-line multiagent chemotherapy. Eleven patients were treated with CHOP and CHOP-like regimens, eight with modified LSG15, and one with hyper-CVAD. In the lymphoma group, eight patients received CHOP and CHOP-like regimens, while the remaining four received LSG15. In the acute group, four patients received LSG15, three CHOP and CHOP-like, and one hyper-CVAD. Intrathecal prophylaxis was administered to 16 patients, while 9 patients received antiretroviral treatment (zidovudine and interferon) alongside chemotherapy. No biological agents were used, as they are not available for ATLL patients in our country.

Second-line chemotherapy was administered to eight patients—to seven patients because of disease progression during first-line treatment and one patient because of relapse after obtaining a complete response. The most frequently used regimens were platinum-based (GEMOx, DHAOx, DHAP)—five patients, followed by hyper-CVAD—two patients and modified LSG15—one patient. Five patients received the third line: four patients who experienced disease progression during the second line and one patient who relapsed after achieving a complete response.

Two lymphoma-type ATLL patients who achieved a complete response underwent allogeneic stem cell transplantation.

The treatment response was assessed by physical examination, laboratory (complete blood cell count, biochemistry studies), and imaging studies. We used the Japan Clinical Oncology Group response criteria [20] to assess diseases response, which we classified as complete response (CR), partial response (PR), stable disease (SD), and progressive disease (PD). Response could not be evaluated in three patients who had a short follow-up period.

Statistical analysis was performed using IBM SPSS Statistic version 25 (IBM Corp., Armonk, NY, USA). Demographics, clinical, laboratory, and treatment data were summarized using descriptive statistics. Categorical variables were summarized as numbers and percentages. We used Fisher’s exact test to compare the differences in the frequency of categorical variables. Continuous variables were summarized as medians with the corresponding ranges or 95% confidence intervals. We used the Independent Samples Median Test to compare the continuous variables. Survival estimates were calculated by Kaplan–Meier and compared using the log-rank test. We compared overall survival (OS) between ATLL subtypes, laboratory findings at diagnosis, JCOG-PI, treatment modalities, and type of response to chemotherapy. Follow-up duration was calculated from ATLL diagnosis to the date of death or last follow-up. Statistical significance was established for *p* < 0.05.

## 3. Results

### 3.1. Demographic and Clinical Features

This study includes 20 patients diagnosed with aggressive-type ATLL, 8 (40%) with acute-type ATLL, and 12 (60%) with lymphoma-type ATLL. The median age at diagnosis was 40.50 years, higher in the acute-type, but the difference was not statistically significant (*p* = 0.650). Most patients were female, with a male-to-female ratio of 1:2.33 (*p* = 0.455).

Upon diagnosis, most patients were found to have bone marrow infiltration, lymphadenopathies, hepatomegaly, and splenomegaly. In addition to bone marrow, cutaneous and pulmonary sites were the most common locations for extranodal infiltration. The most frequent comorbidities were infectious, including hepatitis B and C viruses, as well as tuberculosis. All patients tested positive for anti-HTLV-1 antibodies. None of the patients in our study were HIV-infected.

The baseline demographic and clinical characteristics are summarized in Table 1.

### 3.2. Laboratory Characteristics

The white blood cell (WBC) and absolute lymphocyte counts were significantly higher in the acute-type ATLL (*p* = 0.020). Two of the patients had a WBC count over 50,000/mm^3^. None of the patients were diagnosed with moderate or severe anemia, and only one patient had severe thrombocytopenia, which was actually caused by hepatic cirrhosis.

Regarding the biochemistry results, acute-type ATLL patients had significantly higher corrected calcium serum levels than those with lymphoma-type ATLL (*p* = 0.001). We excluded two patients from the analysis for total bilirubin, one with hepatic cirrhosis and one with major thalassemia. As for creatinine levels, we did not find them significant, as they were elevated in only three patients despite reaching statistical significance.

Peripheral blood immunophenotyping by flow cytometry was performed for all acute-type ATLL patients. The typical CD4+ CD8− phenotype was identified in five patients, coexpression of CD4 and CD8 in two patients, and CD4− CD8+ phenotype in only one patient. CD25 was negative in only one patient. 

Laboratory findings at diagnosis are shown in Table 2.

### 3.3. Disease Outcomes

#### 3.3.1. Complications

During follow-up, all patients experienced complications related to the disease or its treatment (Table 3). The most common complications reported were cytopenia (all patients), infections (all patients), symptomatic hypercalcemia, compression syndrome, and extranodal infiltration. Neurological complications were also frequent, more often due to metabolic causes (hypercalcemia, liver, and pancreatic diseases) and CNS infiltration.

#### 3.3.2. Response to Chemotherapy

We assessed the response to therapy in 17 patients. After the first line, four patients achieved CR (all lymphoma-type), and two patients achieved PR (one of whom had acute-type ATLL). We did not observe a better response rate to either regimen. Only one patient out of eight responded to second-line treatment, and none responded to third- or fourth-line treatment.

#### 3.3.3. Survival

**Figure 1:**
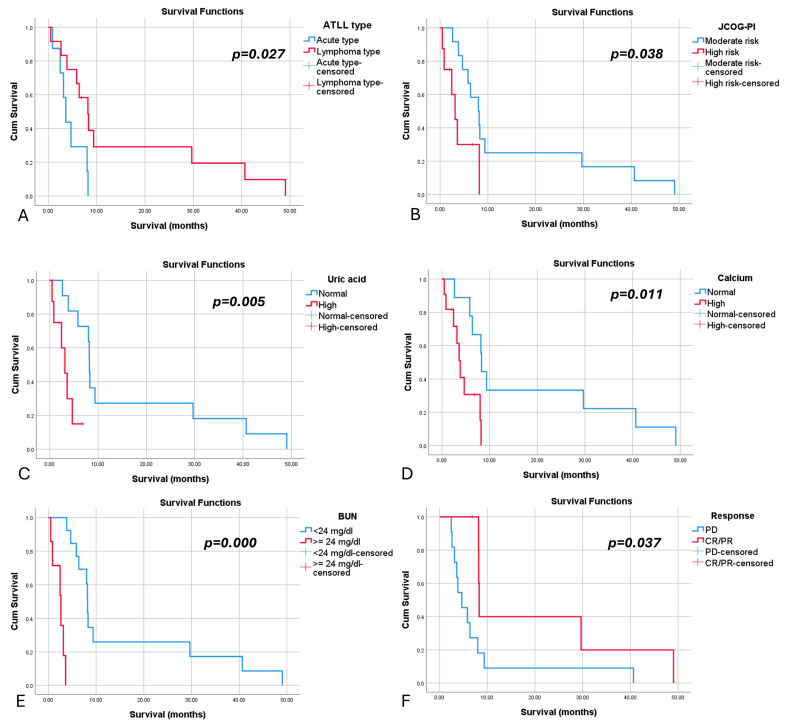
Kaplan–Meier plots for overall survival (OS) in adult T-cell leukemia/lymphoma patients. (**A**). OS by ATLL subtype. (**B**). OS by Japan Clinical Oncology Group prognostic index. (**C**). OS by serum uric acid at diagnosis. (**D**). OS by serum calcium at diagnosis. (**E**). OS by serum blood urea nitrogen (BUN) at diagnosis. (**F**). OS by response to chemotherapy.

The most significant results regarding survival are depicted in Table 4 and Figure 1. Median OS was 6.37 months, with lymphoma-type ATLL showing a higher median OS compared to acute-type ATLL (*p* = 0.027). Hypercalcemia, hyperuricemia, high blood urea nitrogen, high-risk according to JCOG-PI, and lack of achieving CR/PR were associated with statistically significant shorter survival.

## 4. Discussion

Romania is the only European country where HTLV-1 is endemic (5.33 at 10,000 blood donors) [21]. According to our best knowledge, the first case of ATLL in Romania was reported in 1993 [22].

The routes of HTLV-1 transmission are similar to those of HIV. In the 1980s, patients in Romania were infected with HIV due to the reuse of improperly sterilized injection equipment and transfusions with unscreened blood. This group of patients is now known as the “Romanian cohort” [23]. Although HTLV-1 might already have been present in Romania, it likely transitioned into an endemic state in the same period as the HIV outbreak [3].

Despite the high HTLV-1 prevalence in Romania, screening is performed only in blood donors and in the transplant setting. Vertical transmission of HTLV-1 is a well-known route of transmission [24,25,26], having also been reported in one Romanian patient [27]. Screening different population groups, especially pregnant women, could prevent vertical transmission and reduce the burden of HTLV-1-associated diseases. However, to date, there are no systematic screening protocols for pregnant women in Romania.

Our ATLL patients have a lower median age at diagnosis (40.5 years) compared to other countries, possibly due to nosocomial transmission in the 1980s and a higher proportion of vertical transmission. For instance, the median age at diagnosis is 68 years in Japan, 62 years in the United States of America, and 58 years in Latin America [28,29,30]. However, the median age at diagnosis in Romania is closer to that in Brazil (44 years) and Spain (47 years) [31,32].

In our study, we noticed that there was a higher proportion of women compared to men (M:F = 1:2.33). This finding is consistent with results from other studies conducted in Latin America and Spain [30,32]. However, studies carried out in Japan, the United States of America, and Brazil showed either a slight male predominance or no significant difference between genders [28,29,31].

The most common ATLL subtype in our study was lymphoma (60%), followed by the acute subtype (40%). Our findings are similar to those of Latin American and Spanish studies [30,32]. On the other hand, studies from Japan have found that the acute subtype is almost twice as frequent as the lymphoma subtype. Lymphoma-type accounts for 24.9–25.7% of cases, acute-type ATLL for 49.5–51.9%, and the remaining percentages represent the chronic and smoldering subtypes [28,33,34].

Compared to Japanese findings, our patients had a more advanced disease status. We noted a higher proportion of patients with stage III and IV disease (19 out of 20 patients), ECOG ≥ 2 (15 out of 20 patients), and B symptoms (8 out of 11 patients) [28]. Once again, our results were comparable to those from Latin America [30].

Laboratory presentation at diagnosis revealed significant differences between acute-type and lymphoma-type ATLL. Hypercalcemia was one of the most frequent complications of ATLL, and it strongly correlated with acute-type ATLL [28,30]. ATLL patients may experience severe hypercalcemia, a medical emergency defined by a serum calcium level greater than 14 mg/dl and associated with multiorgan dysfunction [35]. In our study, all patients diagnosed with acute-type ATLL had hypercalcemia at diagnosis. Severe hypercalcemia was found in ten patients at diagnosis or during follow-up. Other laboratory findings associated with acute-type ATLL, namely high LDH, hyperuricemia, high total bilirubin, low albumin, and increased blood urea nitrogen, but without reaching statistical significance. These findings align with those reported in previous studies [28,30]. Among laboratory studies, we found that hypercalcemia, hyperuricemia, and high blood urea nitrogen were independent high-risk factors, as previously reported [36].

We identified high-risk patients using JCOG-PI. Most patients in the acute group were characterized as high-risk (six out of eight patients), while most of those in the lymphoma group had moderate-risk (ten out of twelve patients). As expected, survival analysis showed an inferior median OS in the high-risk patients, 3.13 months, compared to 8 months in the moderate-risk group.

Regarding treatment, CHOP, CHOP-like, and modified LSG15 were most frequently used. Mogamulizumab is not available in Romania for ATLL patients. Although a better response rate (CR rate 40% vs. 25%) and higher survival (OS at three years 24% vs. 13%) were reported when patients were treated with LSG15 compared to CHOP [37], we did not obtain any significant differences in our study. This can be explained by the small number of cases as well as the preference of the LSG15 regimen for acute-type ATLL patients, a group that is already linked to a lower response to chemotherapy and a shorter survival.

Even if our patients were younger than the ones in the Japanese studies, overall survival was decreased in our study. Median survival was 3.6 months in our study versus 6.2–8.3 months in Japanese studies for acute-type ATLL, and 8.1 months in our study versus 10.0–10.2 months for lymphoma-type ATLL in Japanese studies [10,28,38]. The survival in our study is similar to the reports from the United States of America, Latin America, and Spain [29,30,32]. In our study, only six patients (30%) obtained CR or PR after first-line treatment, while the response rate in Japan was higher (acute-type: CR 14.3%, PR 49.3%; lymphoma-type: CR 22.3%, PR 45.1%) [28]. Lack of efficient screening strategies, diagnosis in a more advanced stage, and the unavailability of novel therapies such as Mogamulizumab, Ranimustine, and Vindesine limited experience with this rare disease, or possibly unknown disease particularities could be some of the reasons for the worse outcomes of our patients.

We recognize that the similarities between our patients and those from Latin America and Spain, as well as the distinctions from Japanese patients, may also result from infection with various genotypes of HTLV-1 [39,40]. In Romania, the viral strains belong to the Transcontinental subgroup of the Cosmopolitan a-genotype, similar to other parts of Europe and many regions in Latin America [40]. In Japan, strains of viruses from the Transcontinental subgroup coexist with Japanese-specific ones [40].

We acknowledge the limitations of our study: low statistical power due to the small sample size, retrospective collection of data, and limited access to some important laboratory analyses, such as the soluble IL-2 receptor and genetics exam. However, given the low incidence of ATLL, we consider that the current sample size is appropriate in order to detect significant conclusions.

## 5. Conclusions

ATLL is a very aggressive T-cell neoplasm with limited and ineffective treatment options. The particularities of the ATLL patients diagnosed in Romania in the last 12 years are the lowest median age at diagnosis reported in the literature, according to our knowledge, female predominance, and a higher incidence of lymphoma-type ATLL compared to Japanese findings. Despite the young age at diagnosis, survival was inferior to Japanese studies. The epidemiological data, higher incidence of lymphoma-type ATLL, and survival outcomes were closer to the Latin American and Spanish reports. Regardless of its potential to cause severe illness, HTLV-1 often goes undiagnosed due to a lack of awareness and insufficient screening protocols. There is a critical need for enhanced screening efforts, particularly in regions with high prevalence and among pregnant women. Regular follow-up and access to emerging therapies are essential for improving prognosis of these patients.

## Figures and Tables

**Table 1:** Demographic and clinical characteristics of ATLL patients.

Characteristics at Diagnosis	Total (*n* = 20)	Acute-Type (*n* = 8, 40%)	Lymphoma-Type (*n* = 12, 60%)	*p* Value
Age				
median	40.5 y	47.5 y	39.5 y	0.650
(range)	(20–72 y)	(20–72 y)	(35–57 y)	
Sex				
Male	6	3	3	0.642
Female	14	5	9	
Stage				
I–II	1	0	1	1.000
III–IV	19	8	11	
JCOG-PI				
Moderate-risk	12	2	10	0.019
High-risk	8	6	2	
B symptoms				
Yes	8	0	8	0.055
No	3	2	1	
N/A	9	6	3	
ECOG				
0–1	11	2	9	0.065
2–4	9	6	3	
Lymphadenopathies				
Yes	15	4	11	0.109
No	5	4	1	
Hepatomegaly				
Yes	15	8	7	0.055
No	5	0	5	
Splenomegaly	13	6	7	
Yes	13	6	7	0.642
No	7	2	5	
Extranodal involvement				
Bone marrow				0.580
Yes	10	5	5	
No	5	1	4	
N/A	5	2	3	
Skin	3	3	0	0.049
Lung	3	1	2	1.000
CNS	1	0	1	1.000
Digestive tract	1	1	0	0.400
Kidneys	1	0	1	1.000
Parotids	1	0	1	1.000
Sinuses	2	0	2	0.495
Muscles	1	0	1	1.000
Infectious comorbidities				
Hepatitis B virus	2	1	1	1.000
Hepatitis C Virus	3	1	2	1.000
Tuberculosis	4	0	4	0.117

Abbreviations: N/A = not available, CNS = central nervous system, JCOG-PI = Japan Clinical Oncology Group prognostic index, ECOG = Eastern Cooperative Oncology Group performance status.

**Table 2:** Baseline laboratory findings.

Laboratory Tests	Total (*n* = 20)	Acute-Type (*n* = 8)	Lymphoma-Type (*n* = 12)	*p* Value
	Median (Range)	Median (Range)	Median (Range)	
Complete blood cell count				
WBC (/mm^3^)	10,950 (3990–331,620)	33,400 (5600–331,620)	9170 (3990–17,600)	0.020
Neutrophils (/mm^3^)	7445 (3290–36,478)	11,699 (3900–36,478)	7008 (3290–10,620)	0.170
Lymphocytes (/mm^3^)	2350 (330–291,825)	19,100 (1200–291,825)	1291 (330–4256)	0.020
Hemoglobin (g/dl)	13.00 (10.30–16.90)	13.45 (12.10–16.90)	12.45 (10.30–14.70)	0.650
Platelets (/mm^3^)	272,500 (37,000–750,000)	178,500 (37,000–541,000)	291,500 (192,000–750,000)	0.170
Blood biochemistry				
Corrected calcium (mg/dL)	10.32 (8.70–19.64)	12.56 (10.33–19.64)	9.61 (8.70–15.54)	0.001
LDH (U/L)	476.5 (180–2496)	747 (299–2496)	420.5 (180–1791)	0.170
Uric acid (mg/dL)	6.8 (2.1–11.6)	8.1 (3.3–11.6)	4.5 (2.1–8.4)	0.070
Albumin (g/dL)	3.56 (2.20–4.70)	3.45 (2.30–3.80)	3.66 (2.20–4.70)	0.650
Total bilirubin (mg/dL)	0.62 (0.18–24)	1.72 (0.61–24)	0.49 (0.18–1.27)	0.050
ALT (U/L)	39 (11–165)	46.5 (11–100)	34.5 (20–165)	0.650
AST (U/L)	39.5 (17–248)	89 (23–248)	33.5 (17–131)	0.650
Creatinine (mg/dL)	0.80 (0.50–3.40)	1.14 (0.71–3.40)	0.80 (0.50–1.20)	0.005
BUN score (mg/dL)	10.32 (7.93–58.29)	24.50 (12.00–58.29)	12.84 (7.93–28.00)	0.170

Abbreviations: WBC = white blood cell count, LDH = lactate dehydrogenase, ALT = alanine aminotransferase, AST = aspartate aminotransferase, BUN = blood urea nitrogen.

**Table 3:** Most common complications at diagnosis and during follow-up.

Type of Complication	At Diagnosis	During Follow-Up
Infections		
Candida albicans	3	1
Clostridium difficile	0	7
Other bacterial infections	2	10
Herpes zoster	1	0
SARS-CoV-2	0	1
Infections after allo-HSCT		
CMV reactivation	0	2
BK virus	0	1
Symptomatic hypercalcemia	5	5
Compression syndrome		
Mediastinal	3	1
Upper respiratory airways	0	1
Abdominal vessels and extrinsic biliary duct	1	0
Neurological		
Metabolic encephalopathy	2	4
CNS infiltration	1	4
Convulsive seizures due to hyponatremia	0	1
PRES	0	1
Hepatic		
Cytolysis	2	8
Cholestasis	2	5
Acute kidney injury	2	4
Mucositis	0	4
Extranodal infiltration (besides bone marrow)	7	6

Abbreviations: SARS-CoV-2 = severe acute respiratory syndrome coronavirus 2, HSCT = hematopoietic stem cell transplantation, CMV = cytomegalovirus, PRES = posterior reversible encephalopathy syndrome.

**Table 4:** Kaplan–Meier survival analysis of ATLL patients.

Variable	Median Survival (95% CI) (Months)	Log Rank *p* Value
ATLL type		
Acute	3.600 (2.413–4.787)	0.027
Lymphoma	8.167 (5.280–11.053)	
JCOG-PI		
Moderate-risk	8.000 (4.945–11.055)	0.038
High-risk	3.130 (1.416–4.844)	
Extranodal involvement		
No	8.000 (5.107–10.893)	
Yes	4.667 (1.890–7.443)	0.887
Calcium		
Normal	8.300 (7.910–8.690)	0.011
High	3.830 (2.784–4.876)	
LDH		
<2×ULN	9.367 (2.119–16.614)	0.362
≥2×ULN	6.367 (1.759–10.974)	
Uric acid		
Normal	8.200 (7.876–8.524)	0.005
High	3.130 (1.416–4.844)	
BUN		
Normal	8.200 (7.876–8.524)	0.000
High	2.600 (0.681–4.519)	
Albumin		
<3.5g/dL	2.600 (0.825–4.217)	0.694
≥3.5g/dL	8.167 (5.571–10.762)	
Total bilirubin		
Normal	8.000 (4.398–11.602)	0.327
High	3.600 (0.000–9.251)	
Treatment		
CHOP, CHOP-like	4.667 (1.571–7.762)	0.932
LSG15	8.200 (2.121–14.279)	
Response		
CR/PR	8.300 (8.085–8.515)	0.037
PD	4.667 (2.257–7.076)	

Abbreviations: CI = confidence intervals, ATLL = adult T-cell leukemia/lymphoma, ULN = upper limit of normal, CR = complete response, PR = partial response, PD = progressive disease.

## Data Availability

The data presented in this study are available on request from the corresponding author.
